# Essays on Infant Therapeutics

**Published:** 1852-03

**Authors:** 


*Essays on Infant Therapeutics. To which are added, Observations on Ergot, History of the Origin of the use of Mercury in Inflammatory Complaints; together with the Statistics of the Deaths from Poisoning in New York in the years* 1841 – 2 – 3. By JOHN B. BECK, M. D., Prof. of Mat. Med. in the College of Physicians of the University of the State of New York, etc. etc. etc. Second Edition, enlarged and revised. New York: Wm. E. Dean, Publisher, 2 Ann Street. 1852.

The essays by the late lamented Dr. Beck, on Infant Therapeutics, have already been received with much favor by the medical public. The interest in the work will be enhanced by the fact that during the last months of his life, during the intervals of ease permitted by a painful disease, the author prepared the present revised and improved edition. It is calculated to do much good by impressing on the minds of practitioners the importance of employing, with great caution, potent remedial agents in infantile diseases. In this point of view it has probably already exerted a favorable influence on medical practice.

